# Interventions aimed at healthcare professionals to increase the number of organ donors: a systematic review

**DOI:** 10.1186/s13054-019-2509-3

**Published:** 2019-06-20

**Authors:** Marloes Witjes, Nichon E. Jansen, Johannes G. van der Hoeven, Wilson F. Abdo

**Affiliations:** 1 Department of Intensive Care Medicine, Radboud Institute for Health Sciences, Radboud University Medical Center, P.O. Box 9101, Internal post 710, 6500 HB Nijmegen, The Netherlands; 2 Dutch Transplant Foundation, Leiden, The Netherlands

**Keywords:** Organ donation, Organ donor rate, Intensive care, Family guidance, Donor identification, Donor referral, Consent rate

## Abstract

**Background:**

The last decade, there have been many initiatives worldwide to increase the number of organ donors. However, it is not clear which initiatives are most effective. The aim of this study is to provide an overview of interventions aimed at healthcare professionals in order to increase the number of organ donors.

**Methods:**

We systematically searched PubMed, EMBASE, CINAHL, PsycINFO, and the Cochrane Library for English language studies published until April 24, 2019. We included studies describing interventions in hospitals aimed at healthcare professionals who are involved in the identification, referral, and care of a family of potential organ donors. After the title abstract and full-text selection, two reviewers independently assessed each study’s quality and extracted data.

**Results:**

From the 18,854 records initially extracted from five databases, we included 22 studies in our review. Of these 22 studies, 14 showed statistically significant effects on identification rate, family consent rate, and/or donation rate. Interventions that positively influenced one or more of these outcomes were training of emergency personnel in organ donation, an electronic support system to identify and/or refer potential donors, a collaborative care pathway, donation request by a trained professional, and additional family support in the ICU by a trained nurse. The methodological quality of the studies was relatively low, mainly because of the study designs.

**Conclusions:**

Although there is paucity of data, collaborative care pathways, training of healthcare professionals and additional support for relatives of potential donors seem to be promising interventions to increase the number of organ donors.

**Trial registration:**

PROSPERO, CRD42018068185

**Electronic supplementary material:**

The online version of this article (10.1186/s13054-019-2509-3) contains supplementary material, which is available to authorized users.

## Background

The large gap between organ donor availability and organ demand is a major healthcare issue worldwide. In 2017, the Netherlands had 15.2 actual deceased organ donors per million population (PMP), while at the end of 2017, there were still 1138 patients awaiting a transplant and 140 patients who died while on the waiting list [1]. The UK had 22.5 deceased organ donors PMP, 6739 patients awaiting a transplant and 436 patients died while on the waiting list. For the USA, 31.7 deceased organ donors PMP, 77,115 patients were on a transplantation waiting list, and 6021 patients died while on the waiting list [1]. To amend the large organ donor shortage and increase organ donation rates, many initiatives have been suggested. These initiatives range from changing the legal consent system (opt-in versus opt-out) [2], large-scale public campaigns to raise awareness [3], to interventions aimed at increasing the organ donation pool through expanding medical criteria [4]. Due to the continuing shortage, new interventions are proposed frequently.

Many studies have also been published on improving the donation process in the hospital [5]. The donation process starts with the identification of a potential organ donor. Subsequently, the potential organ donor must be referred to the intensive care unit (ICU), if not already admitted, and the organ procurement organization (OPO). In addition, irrespective of the legal consent system, consent must be obtained either by the donor him- or herself via the Donor Registry and/or by family members. Healthcare professionals play an important role in the donation process as they are directly involved and responsible for identifying and referring potential organ donors and obtaining consent. Several studies have shown that potential organ donors are not always recognized, especially when patients die outside the ICU [6–8]. For example, a study from the Netherlands described that the number of unrecognized organ donors outside the ICU was 11–34% of the known organ donor pool [6, 9].

The objective of this study was to pinpoint effective interventions that were aimed at healthcare professionals and had the goal of increasing the number of organ donors.

## Methods

A systematic review of the literature was performed. The criteria for article inclusion and data analysis were pre-specified. The initial protocol has been registered in PROSPERO, the international prospective register of systematic reviews with registration number CRD42018068185 [10].

### Data sources and searches

PubMed (including MEDLINE), EMBASE, CINAHL, PsycINFO, and Cochrane Library were searched until April 24, 2019, restricted to English language publications. The search strategy included the following concepts: post-mortem organ donation, healthcare professionals, and interventions in hospitals. The complete search strategy for each database is presented in Additional file 1. The author’s personal files and references of included studies were also searched to identify additional relevant articles (snowballing).

### Selection criteria and process

Titles and abstracts retrieved from the search strategy were independently screened by two authors (MW and NJ), to identify studies that potentially fulfilled inclusion criteria. Full-text articles were screened by the same two authors. Disagreement on inclusion was resolved by discussion. Studies were included when they met all the following inclusion criteria:

1. The healthcare intervention was aimed at healthcare professionals who were involved in the identification, referral, and support of (relatives of) potential organ donors.
2. Study design was experimental, quasi-experimental, or observational, such as randomized controlled trials, (un) controlled before-after studies, and (non-) controlled cohort studies.
3. Study had at least one quantitative outcome measure.
4. English language full-text article is available.

Studies were excluded when the interventions aimed to increase the potential donor pool or improve logistics outside the hospital, e.g., implementation of a donation after circulatory death (DCD) protocol, implementation of a donation program with transplant coordinators and regional retrieval teams, expanding the donor pool (older donors, DCD donors, non-ventilated donors), education of the population, ways to recover organ function, legislative measures, and improved allocation algorithms. In addition, we excluded donor programs consisting of more than two interventions. Although a bundle might be interesting as an approach, the effect per individual intervention cannot be distinguished. Also, a bundle would mean all components of the bundle have to be implemented to obtain the effect making it more difficult to implement in daily practice.

### Data extraction and quality assessment

One researcher (MW) extracted the data from the included studies, using a standardized form (see Additional file 2). The extracted data were checked by a second researcher (NJ). The extracted data included the study design, objective and methods, setting, population and sample size, intervention, outcomes and results, conclusion, and article comments from the reviewers.

The quality of the included articles was assessed using the suggested risk of bias criteria for EPOC (Effective Practice and Organisation of Care) reviews from the Cochrane Handbook [11]. The criteria for studies with a separate control group (randomized trials, non-randomized trials, and controlled before-after studies) were different from the criteria for studies without a control group (uncontrolled before-after studies, cohort studies). Quality criteria were independently assessed by two authors (MW and NJ). Discrepancies were resolved by discussion between these two authors.

### Data synthesis and analysis

We tabulated study characteristics and outcomes such as study design, intervention, number of participants, outcomes, and significance level. The interventions were described in more detail and classified in one or more of the following categories: (1) identification and referral of the potential organ donor, (2) education of the healthcare professionals, and (3) extra support of the relatives to help them make a well-considered decision on donation.

## Results

**Fig. 1:**
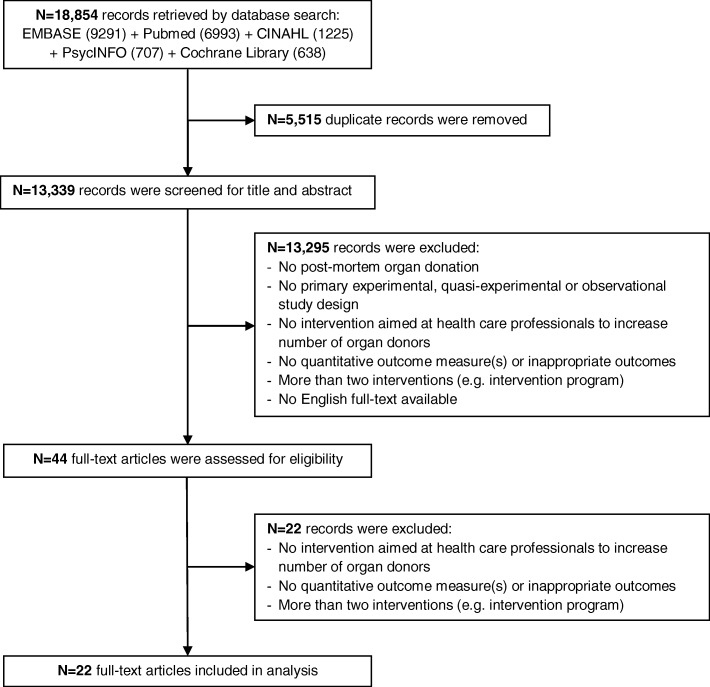
Flow chart showing the inclusion of articles

Our search identified 18,854 records, of which 5515 duplicate records were removed (Fig. 1). In total, 13,339 records were screened for title abstract. After excluding 13,295 records, 44 full-text articles were assessed for eligibility. The final set of articles consisted of 22 full-text articles. No new articles were identified through snowballing.

### Characteristics of included studies

Table 1 summarizes study characteristics and outcomes of the 22 studies. Fourteen uncontrolled before-after studies (UBAs), four cohort studies, two randomized controlled trials (RCTs), one controlled before-after study (CBA), and one non-randomized controlled trial (NRCT) were analyzed. The interventions were aimed at healthcare professionals: physicians, nurses (specialized in donation), requestors, personnel from the procurement centers, social workers, chaplains, administrators, and psychologists. Ten studies were single-center studies, and 12 studies were performed across multiple hospitals ranging from 3 to 220 hospitals. The study population consisted of relatives of ICU patients, potential/eligible donors, emergency department (ED) deaths, declared brain deaths, donation requests, and patients meeting trigger criteria. The sample size per study ranged from 11 to 1101 in the intervention group and from 3 to 1563 in the control group. The majority of the cases were DBD and most studies included donation after brain death as well as DCD, depending on whether the country had a DCD protocol.

**Table 1:** Summary of study characteristics and outcomes

Study, year, country [reference]	Study design	Intervention and setting	Population, n		Intervention(s) vs. control(s)	Outcome	Effect			*p* value
			Intervention	Control			Intervention	Control	Additional group	
Adanir et al. 2014, Turkey [12]	RCT	Psychological support for relatives of patients at one general ICU	First-degree relatives of 100 ICU patients	First-degree relatives of 100 ICU patients	Psychological support vs. no psychological support	Consent rate if patient had become brain dead, %	75	32		< 0.0001
						Consent rate if patient died, %	78	13.9		< 0.0001
Beasley et al. 1997, USA [13]	UBA	A large-scale intervention for physicians, residents, nurses, social workers, chaplains and administrators in 50 hospitals in three OPO service areas	369 potential donors	422 potential donors	After vs. before intervention	Donor identification, %	97.0	90.5		0.001
						Referral rate, %	80.2	55.5		0.001
						Donation requested, %	85.6	69.0		0.001
						Family consent rate, %	52.2	50.9		NS
						Donation rate, %	42.5	32.9		0.005
Beigee et al. 2017, Iran [14]	UBA	More active identification of brain dead cases in hospitals (*n* = NR) affiliated to organ procurement units of Shahid Beheshti University of Medical Sciences	NR	NR	After vs. before intervention: from calling a couple of times per week to calling every day	Reported cases of brain death, n	460	224		NR
						Number of confirmed cases of brain death, n	306	180		NR
						Number of cases transferred to the OPU, n	188	125		NR
						Actual number of donors, *n*	165	115		NR
						Family consent rate, %	90%	75%		< 0.001
						Number of donated organs per each brain-dead case, *n*	2.74	2.67		NR
Bires 1999, USA [15]	Cohort study	An 8-h designated requestor program implemented in one hospital;13 requestors were trained	19 potential donors before, 20 after	9 potential donors before, 15 after	Hospital with designated requestors vs. hospital with organ procurement coordinators	Consent rate before intervention, %	58	66		NR (1.000^a^)
						Consent rate after intervention, %	50	60		NR (0.734^a^)
Bleakley. 2010, UK [16]	UBA	Donor identification scheme and training of 170 staff members in four hospital sites	NR	NR	After vs. before intervention	Number of referrals, *n*	121	4		NR
						Number of successful organ donors, *n*	9	0		NR
						Number of organs transplanted, *n*	22	0		NR
Feest et al. 1990, UK [17]	UBA	Protocol to detect and transfer potential organ donors to the ICU for organ donation implemented in one hospital	18 donors	3 donors	After vs. before intervention	Donors derived outside ICU, *n*	8	0		NR
						Donors from ICU, *n*	10	3		NR
						Possible donors where donation was not discussed, *n*	4	8		NR (0.005^a^: total number of donors from possible donors)
Garside et al. 2012, UK [18]	UBA	An embedded specialist nurse in organ donation (SNOD) and utilization of a collaborative care pathway in one hospital	160 ED deaths	151 ED deaths	After vs. before intervention	Referral to organ donation team from ED, *n*	26	3		< 0.0001
						Patients proceeding to organ donation from ED, *n*	2	0		1.0
						Referral to organ donation team from ICU, *n*	44	9		NR
Henderson et al. 1998, USA [19]	UBA	Educational campaign of emergency personnel in one hospital	1995: 25 potential donors, 1996: 45 potential donors	10 potential donors	One year after intervention (1995) vs. before intervention vs. 2 years after intervention (1996)		1995	1994	1996	1995 vs. 1994
						Referral to OPA from ED, % of potential organ donors	100	10	100	< 0.0001
						Organs procured from ED, *n*	14	0	32	NR
						Actual donors from ED, *n*	NR	0	10	NR
Ismail et al. 2018, Netherlands [20]	Cohort study	A telephone-based advisory support by an experienced trained psychologist for requesters who are about to request for donation.	141 requestors with intervention	1563 requestors without intervention	Intervention vs. control	Consent or assent rate potential donors	58%	35%		< 0.001
						Consent or assent rate potential donors not registered in DR	44%	19%		< 0.001
						Consent rate potential donors who leave decision to next of kin	31%	30%		> 0.99
						Assent rate potential donors registered with permission in DR	93%	91%		0.78
Jansen et al. 2011, Netherlands [21]	NRCT	Nurses were trained in communication about donation and have long-term contact with relatives of potential donors in one hospital	1 hospital (66 relatives)	2 different control hospitals (107 relatives vs. 99 relatives)	Hospital with trained donation practitioners (TDP) vs. control hospital vs. control hospital with hostesses		TDP	Control	Hostess	
						Consent rate including consent in Donor Registry, %	57.5	34.6	39.4	0.003 (TDP vs. control)
										0.022 (TDP vs. hostess)
						Consent rate excluding consent in Donor Registry, %	45.1	21.7	26.3	0.004 (TDP vs. control)
										0.026 (TDP vs. hostess)
						Consent rate organ donation, %	60.0	32.7		< 0.022
Krekula et al. 2014, Sweden [22]	CBA	Donation specialist nurses (DOSSes) who support the local team with the medical care of eligible donors; 7 DOSSes were appointed in a large urban county	96 eligible donors with DOSS participation	15 eligible donors without DOSS participation, 59 before DOSS service	DOSS participated vs. DOSS did not participate vs. before intervention		DOSS	No DOSS	Before	DOSS vs. no DOSS
						Donation rate, %	74	20	37	0.001
						Reason for not becoming actual donors: family vetoes, %	14	60	34	0.001
						Reason for not becoming actual donors: non-willingness deceased, %	7	20	5	NR
Lenzi et al. 2014, Brazil [23]	Cohort study	Requesting donation by OPO professional (intervention), In-Hospital Coordinator (IHC) or ICU physician in Rio de Janeiro, Brazil	167 (2011) and 248 (2012) OPO	63 (2011) and 55 (2012) ICU; 55 (2011) and 108 (2012) IHC	OPO vs. ICU (not trained) vs. IHC		OPO	ICU	IHC	
						Consent rate 2011, %	63.5	12.7	41.8 53.7	NR (< 0.001^a^) NR (< 0.001^a^)
						Consent rate 2012, %	64.5	20.4		
Linyear et al. 1999, USA [24]	UBA	Implementation of a systematic hospital-based program at Virginia Commonwealth University	Post 1997: 27 potential donors Post 1998: 20 potential donors	42 potential donors	After intervention 1997 vs. before intervention vs. after intervention 1998		After 1997	Before	After 1998	
						Referral rate, %	93	95	90	NR (0.734^a^)
						Approach rate, %	93	88	90	NR (0.833^a^)
						Consent rate, %	44	49	72	NR (0.153^a^)
						Donation rate, %	26	36	50	NR (0.235^a^)
Manyalich et al. 2012, international [25]	UBA	Training program implemented in 220 hospitals in 16 countries	1101 declared brain deaths	784 declared brain deaths	After vs. before intervention	Utilized donors identified, mean ± SD (range)	20.0 ± 17.1 (1–78)	15.7 ± 14.3 (2–69)		0.014
						Organs recovered, mean ± (range)	59.3 ± 52.2 (2–247)	49.7 ± 48.6 (0–228)		0.044
Mulvania et al. 2014, Australia [26]	UBA	Customized, self-sustaining training program area in Australia	NR	NR	3 years during implementation (2011–2013). Pilot program started October 2011.		2013	2011	2012	
						Number of deceased brain dead donors, n	391	337	354	NR
						Request rate, %	96	94	92	
						Consent rate, %	62	59	61	NR NR NR
						Conversion rate, %	53	49	51	
Sandiumenge et al. 2018, Spain [27]	UBA	An instant messaging application (WhatsApp@) was implemented in order to refer potential donors to the DC	74 potential donors outside ICU	40 potential donors outside ICU	After vs. before intervention		After	Before		
						Referral of possible donors to DC from outside ICU	62%	32%		< 0.05
						Proportion donors outside ICU from BD donors in hospital	29%	13%		< 0.05
Siminoff et al. 2009, USA [28]	UBA	Training program ‘Communicating Effectively About Donation’ in 17 hospitals	325 eligible donors	134 eligible donors	After vs. before intervention	Consent rate, %	55.5	46.3		0.07
						Time-sensitive referrals, *n* (% of eligible donors)	281 (86.5)	116 (86.6)		0.97
Siminoff et al. 2015, USA [29]	RCT	Online training program ‘Communicating Effectively about Donation’ in 9 OPOs. CEaD1: theoretical. CEaD2: theoretical and practical (Table 1).	CEaD1: 558 requests, CEaD2: 368 requests	677 requests	After CEaD1 vs. before intervention vs. After CEaD2		CEaD1	Before	CEaD2	CEaD1 vs. CEaD2
						Consent rate, %	83	84	86	NS
						Consent rate novice, %	80	78	89	0.03
						Consent rate midlevel, %	76	81	88	0.004
						Consent rate senior, %	92	89	83	0.02
Stark et al. 1994, USA [30]	UBA	Nurse requestor educational program in one hospital; 25 requestors were trained	11 potential donors	15 potential donors	After (1993) vs. before (1991) intervention	Referrals/requests, *n* (%)	11 (100)	10 (67)		NR (0.053^a^)
						Consent/donations, *n* (%)	8 (73)	4 (27)		NR (0.198^a^)
Von Pohle et al. 1996, USA [31]	Cohort study	Decoupled presentation of the option of organ donation by OPO representative in one hospital	34 potential donors	47 potential donors	After vs. before intervention	Donation rate, %	59	38		< 0.05
Young et al. 2009, UK [32]	RCT	Collaborative requesting by potential donor’s clinician and donor transplant coordinator in 79 ICUs in the UK	100 relatives	101 relatives	Collaborative requesting vs. routine requesting by the clinical team alone	Consent rate intention to treat, %	57	62		0.53
						Consent rate per protocol, %	67	60		0.33
Zier et al. 2017, USA [33]	UBA	Implementation of an electronic decision support system to identify patients who meet OPO notification criteria in one hospital	30 patients meeting trigger criteria	58 patients meeting trigger criteria	After vs. before intervention	Time to referral, hours (range)	1.7 h (0–23.2 h)	30.2 h (0–288.5 h)		0.015
						Donor conversion rate, %	9/10 = 90%	6/12 = 50%		0.074
						Proportions of notifications occurring ≤ 1 h, %	70%	36%		0.003
						Median time to notification, hours	< 0.01 h	3.5 h		0.001
						Total organ donors/critical care death, %	11/24 = 46%	7/57 = 12%		0.002

*Abbreviations*: *RCT* randomized controlled trial, *ICU* intensive care unit, *UBA* uncontrolled before-after study, *OPO* organ procurement organization, *NS* not significant, *NR* not reported, *SNOD* specialist nurse in organ donation, *ED* emergency department, *OPA* organ procurement agency, *NRCT* non-randomized controlled trial, *TDP* trained donation practitioner, *DOSS* donation specialist nurse, *CBA* controlled before-after study, *IHC* in-hospital coordinator, *CEaD* communicating effectively about donation

^a^*p* value was not reported in article, but was calculated based on the outcomes and number of participants given

Various outcomes were reported: donor identification, donor referral (from the ED), family approach rate, consent rate, donation rate, and organs recovered. Most interventions were aimed at increasing the referral rate (from the ED), consent rate or donation rate (which is based on the referral and consent rate). Significant differences were seen in all of these three outcomes. In eight studies, the significance level was not reported. If possible, we calculated the *p* value with the data that were available (Tables 1 and 2).

**Table 2:** Overview of the interventions classified in three categories

Study [reference]	Intervention	Relevant actions	Key players	Classification			Significant effects
				Identification and referral	Education	Support of relatives	
Adanir et al. [12]	Psychological support for relatives	The relatives in the intervention group attended interviews every 2 days with a psychologist if they wanted to. At least 3 therapeutic interviews were completed.	Psychologists			X	Yes
Beasley et al. [13]	Hospital adapted interventions	Monitoring of organ donation, implementation strategy, introduction of recommended practices, development of multidisciplinary team.	Physicians, residents, nurses, social workers, chaplains and administrators	X			Yes
Beigee et al. [14]	Donor identification	The procurement centers call every day to ICUs, emergency departments, coronary care unit, neurosurgery and supervisors of medical centers and trauma centers.	Trained personnel from organ procurement centers	X			NR
Bires [15]	Training of requestors	An 8-h designated requestor program was conducted by the OPO.	Requestors		X		NR (no^a^)
Bleakley [16]	Donor identification	Implementation of a donor identification scheme.	Staff members	X			NR
Feest et al. [17]	Donor identification	The protocol describes the criteria of identification of potential organ donors and enables transfer of patients to ICU for ventilatory support until organ retrieval can be arranged.	Physicians, transplant team, representatives of nurses from medical wards, ICU	X			NR (yes^a^)
Garside et al. [18]	Specialist nurse in organ donation (SNOD) and collaborative care pathway	The role of the SNOD involves close liaison with ICU and ED staff at all levels, ensuring a multidisciplinary collaborative approach to the early identification and management of potential donors. A collaborative care pathway was introduced to identify clinical triggers and facilitate the referral of potential organ donors.	SNODs and ICU and ED staff	X		X	Yes
Henderson et al. [19]	Training of emergency personnel	The OPO educated the emergency personnel on the process of identifying potential donors, and the need for early OPO referral. The OPO also visits the ED every 2 to 3 months to reeducate the staff.	Emergency personnel	X	X		Yes
Ismail et al. [20]	Support by a CaD-trained psychologist for requesters	The Communication about Donation Telephone Advice by Psychologist (CaD-TAP) intervention was developed. The CaD-TAP intervention allows the requester to get general practical advice on effective communication from a CaD-trained psychologist shortly before the actual donation request.	Requesters		X		Yes
Jansen et al. [21]	Training of nurses	Nurses completed the training ‘Communication about donation’. The trained donation practitioners are always available, 24 h a day, and guide the relatives through the donation decision process.	Nurses			X	Yes
Krekula et al. [22]	Training of nurses	The donation specialist nurse (DOSS) on call supports the local team with the medical care of the donors and with the actual donation request, primarily together with the local physician. The DOSSes also promote adherence to standard routines concerning organ donation and take responsibility for the follow-up with DR at their local hospitals.	Nurses			X	Yes
Lenzi et al. [23]	Donation request by trained professional	Performances in obtaining informed consent from potential donors’ families were compared according to the type of healthcare professional conducting the interviews: OPO, In-hospital coordinator or ICU physician (not trained).	OPO, in-hospital coordinators, ICU physicians		X		NR (yes^a^)
Linyear et al. [24]	Family communication protocol	A standard family communication protocol was developed to ensure consistent identification of all patients with devastating neurological insults who might progress to brain death, optimal family communication and support, and a request for organ donation in accordance with best-demonstrated practices.	Nurses and physicians from the ICUs, as well as hospital administrators, chaplains, and LifeNet representatives	X		X	NR (no^a^)
Manyalich et al. [25]	Training of healthcare professionals	Three educational initiatives were designed and implemented: 1) essentials in organ donation 2) professional training for junior transplant coordinators and 3) organ donation quality management. A public website, a private virtual platform and an e-learning campus were used as communication tools.	Professionals in ICUs, postoperative recovery, emergency rooms, etc. (in areas where organ donors can be actively detected)		X		Yes
Mulvania et al. [26]	Training of healthcare professionals	A customized, self-sustaining training program. Two 1-day pilot training sessions were provided to 45 Australian donation leaders. Also, 26 2-day family donation conversation workshops were held in 8 cities (646 participants).	Professionals from the Australian DonateLife Network, ICU, and emergency specialists		X		NR
Sandiumenge et al. [27]	Donor identification and referral	Ninety percent of the specialists playing a key role in the management of possible donors outside the ICU were voluntarily included in a virtual collaborative group using an instant messaging application (WhatsApp@) in order to refer to the DC all patients presenting with GCS < 9 and who fulfilled any of the established by consensus criteria.	Professionals playing a key role in the management of possible donors outside the ICU	X			Yes
Siminoff et al. [28]	Training of healthcare professionals	The training was divided into a day-long interactive group workshop, taught by the principal investigator and then individual skills-based simulated donation scenarios with feedback.	OPO staff members		X		No
Siminoff et al. [29]	Online training of healthcare professionals	Two versions were developed: 1) CEaD1: requesters viewed a series of 4 donation scenarios of increasing difficulty embedded within a web-based tutorial. An accompanying workbook detailed the specific skills needed to effectively initiate request, etc. 2) CEaD2: requesters received the same training as CEaD1, together with live practice and feedback using simulated family scenarios.	Requesters		X		Yes
Stark et al. [30]	Training of nurse requestors	The education program was designed to encompass four concepts: awareness, recognition, offering the option of donation and bereavement.	Nurse requesters	X	X		NR (no^a^)
von Pohle [31]	Donation request by OPO representative	Institution started working with a dedicated representatives from the local OPO who uses decoupling routinely. They spend whatever amount of time is needed with the family to explain the process of donation.	OPO representatives			X	Yes
Young et al. [32]	Collaborative requesting	Collaborative requesting by clinician and donor transplant coordinator.	Clinician and transplant coordinator			X	No
Zier et al. [33]	Donor identification	An Electronic Decision Support system was developed to identify patients who meet OPO notification criteria impending brain death. When the algorithm detects a patient who fulfills notification criteria, a system-generated email is sent directly to the OPO.	OPO	X			Yes

*Abbreviations*: *ICU* intensive care unit, *NR* not reported, *OPO* organ procurement organization, *SNOD* specialist nurse in organ donation, *ED* emergency department, *DOSS* donation specialist nurse, *CEaD* communicating effectively about donation

^a^*p* value was not reported in article, but was calculated based on the outcomes and number of participants given

### Methodological quality

The results from the quality assessment are shown in Additional file 3. Overall, the quality of the studies was relatively low, mainly because of the study designs that were used. Seventeen studies did not use a control group, which makes the criterion “intervention independent of other changes” for these studies at high risk. Two RCTs were performed [12, 32]. One of these studies did not use a correct randomization method. They divided the groups by even and odd numbered beds [12]. In addition, most studies lacked adequate power or had selection bias leading to a high-risk score on the criterion “other risks of bias.”

The studies found in this systematic review were not suitable for a meta-analysis due to heterogeneity in interventions and outcome, the different definitions used for “potential donor” [34, 35], and different (legal) systems used in every country.

### Effects of the interventions

Table 2 provides an overview of the interventions identified in the 22 articles, classified in 3 categories of interventions: identification and referral, education, and extra support of the relatives. Some interventions could be classified in more than one category.

#### Identification and referral

Ten studies focused on the identification and referral of potential organ donors [13, 14, 16–19, 24, 27, 30, 33]. Six out of ten studies focused on donor identification and referral [13, 14, 16, 17, 27, 33]. Two of these six studies showed statistically significant higher identification rates [13, 33] after the intervention. In the study by Beasley et al. [13], a multidisciplinary strategy was introduced in 50 hospitals which increased the donor identification from 90.5 to 97.0% (*p* = 0.001) and the donation rate from 32.9 to 42.5% (*p* = 0.005). In the study by Zier et al. [33], the donation rate increased from 12 to 46% by implementing an electronic decision support system to identify potential organ donors (*p* = 0.002). The study by Sandiumenge et al. [27] used technology to refer potential organ donors to the donation coordinator (DC). Ninety percent of the specialists playing key role in the management of possible donors outside the ICU were included in a WhatsApp group in order to refer to the DC. After the intervention, 62% of the possible donors outside the ICU were referred to the DC compared to 32% before the intervention (*p* < 0.05). These referred donors after the intervention had a mean age of 72 years, and the main cause of death was hemorrhagic stroke (59%) followed by ischemic stroke (33%). Three of the six studies which focused solely on donor identification showed an increase in the number of referrals; however, it was not reported whether this was statistically significant [14, 16, 17]. The studies of Bleakly et al. [16] and Feest et al. [17] focused on donor identification by implementing an identification scheme describing the criteria of identification for personnel in the ED. In the study by Beigee et al. [14], procurement centers called the hospital departments every day to check if there were any potential donors. This resulted in an increase in the number of brain death cases that were transferred to the OPU from 125 to 188. The mean age of these 188 donors was 45 years. In most cases, the cause of brain death was a cerebrovascular accident (47%).

Four out of ten studies, focusing on identification and referral, also focused on education [19, 30] or support of relatives [18, 24]. The study by Henderson et al. [19] showed that a training for emergency personnel on the process of identifying potential donors significantly increased the referral rate from 10 to 100% and the number of actual donors from 0 to 10 (*p* = not reported). A nurse requester education program led to an increased donation rate, however not statistically significant [30]. In the study by Garside et al. [18], an embedded specialist nurse in organ donation (SNOD) and a collaborative pathway was introduced to identify clinical triggers and facilitate the referral of potential organ donors. This led to an increase in referral from the ED from 3 to 26 (*p* < 0.0001). It did, however, not lead to a significant increase in organ donors from the ED (0 to 2). The family communication protocol that was introduced in the study by Linyear et al. [24], did not lead to an increased referral rate, but showed a non-significant increase in donation rate from 36 to 50% in 2 years after the introduction (*p* = 0.235).

#### Education of healthcare professionals

In total, nine studies focused on education of healthcare professionals [15, 19, 20, 23, 25, 26, 28–30]. A cohort study by Ismail et al. [20] showed that advisory support by a trained psychologist for requesters who are about to request for donation increased the family consent rate from 35 to 58% (< 0.001). A retrospective cohort study by Lenzi et al. [23] showed that when family conversations were done by an OPO representative or in-hospital coordinator, this led to significantly higher consent rates than when this was done by non-trained ICU physicians (respectively 64.5%, 53.7%, and 20.4%). In the study by Manyalich et al. [25], an advanced training program was implemented in 220 hospitals in 16 countries, which was adapted to the country’s needs. This training program consisted of three educational initiatives: essentials in organ donation, professional training for junior transplant coordinators, and organ donation quality management. Online communication tools were used to implement these initiatives. The results of this study showed an increase in the mean number of utilized donors identified from 15.7 to 20.0 (*p* = 0.014) and the mean number of organs recovered from 49.7 to 59.3 (*p* = 0.044). Siminoff et al. [28] designed a training program for OPO staff members consisting of a day-long interactive workshop and individual skills-based simulated donation scenarios with feedback. The training led to an increase in consent rate from 46.3% to 55.5% (*p* = 0.07). This study was followed by another study by Siminoff et al. [29], where two online versions of the training program were developed. Overall, this did not lead to an increase in consent rate (84% before intervention, 83% after intervention).

#### Additional support of relatives

Seven studies focused on additional support of relatives [12, 18, 21, 22, 24, 31, 32]. Six out of seven studies showed a statistically significant increase in the main outcome measure after the intervention. In the study by Adanir et al. [12], psychologists performed therapeutic sessions with the relatives of ICU patients. Although the relatives were not relatives of potential organ donors, the percentage of people that would consent to donation, if they had to decide, was higher in the intervention group (75%) than the control group (32%) with *p* < 0.0001. In the study by Jansen et al. [21], nurses were trained who were available 24 h a day to guide the relatives of potential donors. A significantly higher consent rate was seen in the intervention hospital with the trained nurses (57.6%), than in the two control hospitals (34.6% and 39.4%). The mean age of the potential donors in the intervention group was 63 years. The study by Krekula et al. [22] also showed an increase in donation rate when working with trained nurses (called “donation specialist nurse” (DOSS)), first in a DOSS local project and later in a DOSS county-based service (Table 1 shows the outcomes of the DOSS service). In the ACRE trial [32], it was shown that collaborative requesting by a clinician and donor transplant coordinator did not increase the consent rate when compared to requesting by the clinical team alone (57% vs. 62%, *p* = 0.53).

**Fig. 2:**
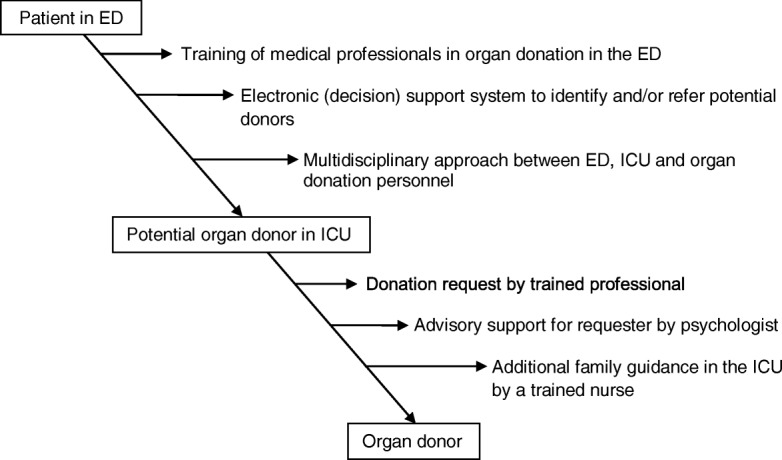
Areas with beneficial interventions focused on healthcare professionals. *Abbreviations*: ED, emergency department, ICU intensive care unit

In Fig. 2, the beneficial interventions are summarized and visually displayed per area.

## Discussion

This systematic review provides an overview of interventions aimed at healthcare professionals in order to increase the number of organ donors. Interventions, with statistically significant effects, were found in all three categories: identification and referral, education, and extra support of relatives, where some interventions focused on two categories. However, the results we found were based on studies with a relatively low methodological quality. Also, many of the included studies were with insufficient power. The lack of high-quality studies seems typical for the research area and for research that is being developed and implemented in practice.

We found that implementation of a collaborative approach between the ICU and other departments, such as the department of neurology, ED, and neurosurgery (“collaborative care pathway”) to identify triggers, facilitates identification and referral of potential organ donors [16, 18, 24]. Implementing such a collaborative care pathway creates the possibility to make organ donation part of end-of-life care, also outside the ICU. Recently, more literature, including studies that were not included in this systematic review, has been published on this topic [5, 6, 36–39]. A recent paper by Martinez-Soba et al. described their experience with an Intensive Care to facilitate Organ Donation (ICOD) protocol [38]. They retrospectively reviewed patients with a devastating brain injury whose families were approached to discuss the possibility of ICOD. This also included patients in which the decision was made not to intubate (50% of the cases). The results showed that ICOD was well accepted by families and ICOD contributed to 33% of the actual donors. Another recent paper by Witjes et al. describes their experience with the implementation of a multidisciplinary approach in the ED. They describe that organ donors from the ED with a fatal brain injury are an important portion (29%) of the total pool of organ donors. Although such an intervention is not straightforward to implement due to its multidisciplinary approach, it could lead to improved donation awareness and better donor identification in the ED.

In some studies, a large number of referrals did not lead to effected organ donors. For instance, in the study by Bleakley et al. [16], it was shown that an increase in referrals to the on-call donor transplant coordinator from 4 to 121 referrals led to an increase in organ donors from 0 to 9. This means that per organ donor, 13 referrals to the OPO were needed (donation rate of 7.4%). A difficulty in analyzing such data is that not each referred patient was actually a potential organ donor and that there are international differences between the definition of a potential organ donor [34, 35].

With regard to the organ donation request, most studies showed that the professional requesting donation should be trained, although not all studies showed a significant effect, mostly due to low sample size [15, 28, 30]. The person who is requesting for donation also differs per country. For example, in the USA, the OPO (who is also involved in the transplant side) is requesting for donation, and in the UK, it is the SNOD who is requesting for donation (and is not involved in the transplant side). In many countries, the requester is part of the treating team and is mostly an ICU physician.

Much research has been published on factors in the donation process that influence consent rates [40–48]. This research also showed that the skills of the requester influence the consent rate, just as the information discussed during the request, understanding of brain death, timing of the request, setting in which request is made, characteristics of the requester, the family ‘s satisfaction level with the medical attention, et cetera. All this information was used to develop various educational programs for healthcare professionals involved in donation practices, some of them shown in our review. In addition, in a large nationwide study including 1322 organ donation requests, it was recently shown that when the requesting physician contacted the OPO before the organ donation request and discussed the case, this led to a higher consent rate [48]. This was presumably because the contact between requesting physician led to more clarity in the conversations with the family as the requesting physician could provide more specific information regarding (suitability for) donation and approximation of the time span of logistics surrounding organ donation.

With regard to family guidance, we found that additional support of relatives by healthcare professionals increased the consent and donation rates. The healthcare professionals were mostly nurses who were trained in organ donation in order to support the relatives of potential organ donors in their decision-making process. On the other hand, collaborative requesting by clinician and donor transplant coordinator did not increase consent rate [32].

### Limitations

Our systematic review has several limitations. First, the studies included in our review are mostly uncontrolled before-after studies which tend to overestimate the effect. On the other hand, these study designs are more feasible in practice than randomized controlled trials, since blinding and concealment of allocation are often not achievable in this area. Second, we did not include articles that reported effects of combined interventions as the effect per individual intervention could not be distinguished, making it harder to implement such an intervention in the daily practice. An earlier review [49] (with articles until 2010) on interventions aimed at healthcare professionals, did evaluate these donor programs with combined interventions and found that the evidence of the 15 included articles was weak due to methodological flaws, as a vague definition of the intervention, lack of explanations on the study design, and unjustified sample size. Third, we only included full-text articles available in English, which may have increased the risk of publication bias. Fourth, many of the included articles dated back to > 20 years ago. This could make their data less applicable to the current practice.

The paucity of data in peer-reviewed journals does not mean that there is no evidence for successful initiatives to increase the number of organ donors. Although not the focus of our review, there have been successful donor programs (with combined best practices) that have improved and sustained organ donation [50, 51]. The focus on potential organ donors outside the ICU, e.g., ED, could be an area where a collaborative effort between the ICUs and ED can increase the number of organ donors and more data is needed from successful collaborative efforts [6, 37–39]. Besides scientific evidence, other (policy) documents exist on interventions that could increase the number of potential organ donors [52]. However, much of these data are not published in peer-reviewed journals, which makes them more difficult to assess and compare to scientific standards. We would like to make a call to action to research, audit, and evaluate initiatives to improve organ donation practices, and to publish these results in scientific papers.

### Recommendations and future research

Based on our extensive literature search, the following recommendations can be made.

With regard to the identification and referral of potential organ donors in the ED, we recommend that hospitals develop a process that ensures that all potential organ donors are identified. Most hospitals will already have such a systematic approach for patients in the ICU. However, such a systematic approach is mostly lacking for potential organ donors outside the ICU, e.g., the emergency department. Successful approaches focusing on this area included a close collaboration between the organ procurement staff, the ICU and departments involved outside the ICU such as the emergency department, the department of neurology, neurosurgery and traumatology. In addition, educating medical professionals outside the ICU in organ donation is paramount in such an approach. Important questions in such a collaborative approach are “who should make the organ donation request?” “where should the organ donation request be made (ED or ICU)?” “what logistical arrangements are required (and should be arranged beforehand) to admit potential donors to the ICU?”

Concerning the consent rate, it is important that the professional who is requesting for donation should be trained in communicating organ donation. In addition, ICU nurses could play an important role in guiding the family during and after the consent process.

It is known that clear communication and information about the donation process are crucial for the family. The role that ICU nurses or other professionals could play in family guidance needs further research as the scientific evidence is limited. Future research could also focus on technology that could be used to (automatically) identify and refer potential organ donors Although randomized controlled trials are difficult to perform in this area, it is important that future research studying new interventions also include control groups. Ideally, a control group is compared to an intervention group in the same time period and prospectively measured.

## Conclusions

In conclusion, this systematic review describes interventions that lead to higher numbers of organ donors. The main finding is that collaborative care pathways, in which donor identification criteria are identified, training of healthcare professionals (also in the ED) and additional focus on support of relatives of potential donors, could be promising interventions to increase the number of organ donors. The paucity of data in peer-reviewed journals asks for a call to action to publish the results of initiatives to improve organ donation.

## Additional files

Additional file 1: Search strategy for each database. This additional file shows the search strategies that were used in the different databases. (DOC 42 kb)

Additional file 2: Data extraction form. This extraction form was used to extract the data from the included articles. (DOC 246 kb)

Additional file 3: Quality assessment of the included studies according to suggested risk of bias criteria for Effective Practice and Organisation of Care (EPOC) reviews [11]. This additional file shows the quality assessment of the included studies. (DOCX 16 kb)

## Data Availability

The datasets used and/or analyzed during the current study are available from the corresponding author on reasonable request.

## Abbreviations

CBA: Controlled before-after study
DC: Donation coordinator
DCD: Donation after circulatory death
DOSS: Donation specialist nurse
ED: Emergency department
EPOC: Effective Practice and Organisation of Care
ICOD: Intensive Care to facilitate Organ Donation
ICU: Intensive care unit
NRCT: Non-randomized controlled trial
OPO: Organ procurement organization
PMP: Per million population
RCT: Randomized Controlled Trial
SNOD: Specialist nurse in organ donation
UBA: Uncontrolled before-after study
