# Supplementary material for: Interventions aimed at healthcare professionals to increase the number of organ donors: a systematic review
Source: Crit Care. 2019 Jun 20;23:227. doi: 10.1186/s13054-019-2509-3 (PMC6587298; doi:10.1186/s13054-019-2509-3)
Supplement: Supplementary file 1 — Search strategy for each database. This additional file shows the search strategies that were used in the different databases. (DOC 42 kb) [file 13054_2019_2509_MOESM1_ESM.doc]

**Additional file 1.** Search strategy for each database

**Pubmed until April 24, 2019**

(Tissue and Organ Procurement [Mesh] OR Brain Death [Mesh] OR Organ donor*[tiab] OR Potential donor*[tiab] OR Organ donat* [tiab] OR Organ procurement*[tiab] OR Brain death[tiab] OR Brain dead[tiab]) AND (Patient Care Team [Mesh] OR Critical Care Nursing [Mesh] OR Medical staff [Mesh] OR Nurses [Mesh] OR Nursing staff [Mesh] OR Physicians [Mesh] OR Professional-family relations [Mesh] OR Attitude of Health Personnel[mesh] OR Critical care[mesh] OR Intensive care units[mesh] OR "Terminal Care"[Mesh:NoExp] OR Patient care [tiab] OR Medical staff [tiab] OR Physician* [tiab] OR Health personnel[tiab] OR Nurse* [tiab] OR Critical Care [tiab] OR Intensive care [tiab] OR Terminal care[tiab] OR End-of-life care[tiab] OR Hospital*[tiab] OR Requestor*[tiab] OR Requester*[tiab] OR Organization* [tiab] OR Organisation* [tiab] OR Coordinator* [tiab] OR Professional*[tiab]) AND (Communication [Mesh] OR Cooperative Behavior [Mesh] OR Hospital planning / organization and administration [Mesh] OR Education, Professional [Mesh] OR Program development [Mesh] OR Referral and consultation [Mesh] OR Efficiency, organizational [Mesh] OR Critical Pathways [Mesh] OR Third-party consent [Mesh] OR Quality of Health Care [Mesh] OR Health Services Research[mesh] OR Interinstitutional Relations[mesh] OR Intervention* [tiab] OR Strateg* [tiab] OR Behavior [tiab] OR Behaviour [tiab] OR Practice* [tiab] OR Protocol* [tiab] OR Procedure* [tiab] OR Structure* [tiab] OR Attitud* [tiab] OR Evaluat* [tiab] OR Training [tiab] OR Education [tiab] OR Communication [tiab] OR Request* [tiab] OR Program [tiab] OR Programme[tiab] OR Planning [tiab] OR Management* [tiab] OR Identif* [tiab] OR Referral* [tiab] OR authorization [tiab] OR authorisation [tiab] OR Consent* [tiab] OR Decision* [tiab] OR Efficacy [tiab] OR Efficiency [tiab] OR Satisfaction [tiab] OR Knowledge [tiab] OR Skill* [tiab] OR Quality[tiab])

Hits: 6993.

**EMBASE until April 24, 2019**

(organ donor/ OR (Organ donor* OR Potential donor* OR Organ donat* OR Organ procurement* OR Brain death OR Brain dead).ti,ab,kw.) AND (Intensive care nursing/ OR Medical staff/ OR medical specialist/ OR exp nurse/ OR nursing staff/ OR physician/ OR anesthesist/ OR emergency physician/ OR hospital physician/ OR intensivist/ OR neurologist/ OR human relation/ OR doctor patient relation/ OR nurse patient relationship/ OR health personnel attitude/ OR nurse attitude/ OR physician assistant attitude/ OR physician attitude/ OR intensive care/ OR intensive care unit/ or terminal care/ OR (Patient care or Medical staff OR Physician* OR Health personnel OR Nurse* OR Critical Care OR Intensive care OR Terminal care OR End-of-life care OR Hospital* OR Requestor* OR Requester* OR Organization OR Organisation OR Coordinator).ti,ab,kw.) AND (interpersonal communication/ OR exp cooperation/ OR hospital planning/ OR hospital management/ OR hospital organization/ OR staff training/ OR medical education/ OR program development/ OR patient referral/ OR "organization and management"/ OR clinical pathway/ OR informed consent/ OR exp health care quality/ OR health care planning/ OR health services research/ OR health service/ OR emergency health service/ OR family service/ OR public relations/ OR (Intervention* OR Strateg* OR Behavior OR Behaviour OR Practice* OR Protocol* OR Procedure* OR Structure* OR Attitud* OR Evaluat* OR Training OR Education OR Communication OR Request* OR Program OR Programme OR Planning OR Management* OR Identif* OR Referral* OR authorization OR authorisation OR Consent* OR Decision* OR Efficacy OR Efficiency OR Satisfaction OR Knowledge OR Skill* OR Quality).ti,ab,kw.)

Hits: 9291.

**CINAHL until April 24, 2019**

((MH "Organ Procurement+") OR (MH "Brain Death") OR TI Organ donor* OR AB organ donor* OR TI Potential donor* OR AB potential donor* OR TI Organ donat* OR AB organ donat* OR TI Organ procurement* OR AB Organ procurement* OR TI Brain death OR AB Brain death OR TI Brain dead OR AB brain dead) AND ((MH "Multidisciplinary Care Team") OR (MH "Critical Care") OR (MH "Medical Staff+") OR (MH "Nurses") OR (MH "Nursing Staff, Hospital") OR (MH "Physicians") OR (MH "Physicians, Emergency") OR (MH "Neurologists") OR (MH "Professional-Family Relations") OR (MH "Attitude of Health Personnel") OR (MH "Physician Assistant Attitudes") OR (MH "Physician Attitudes") OR (MH "Nurse Attitudes") OR (MH "Intensive Care Units") OR (MH "Terminal Care") OR (MH "Palliative Care") OR TI Patient care OR AB Patient care OR TI Medical staff OR AB Medical staff OR TI Physician* OR AB Physician* OR TI Health personnel OR AB Health personnel OR TI Nurse* OR AB Nurse* OR TI Critical Care OR AB Critical Care OR TI Intensive care OR AB Intensive care OR TI Terminal care OR AB Terminal care OR TI End-of-life care OR AB Hospital* OR TI Requestor* OR AB Requestor* OR TI Requester* OR AB Requester* OR TI Organization OR AB Organization OR TI Organisation OR AB Organisation OR TI Coordinator OR AB Coordinator ) AND ((MH "Communication+") OR (MH "Communication Barriers") OR (MH "Communication Skills") OR (MH "Cooperative Behavior") OR (MH "Hospital Planning") OR (MH "Education, Medical+") OR (MH "Program Development+") OR (MH "Referral and Consultation") OR (MH "Organizational Efficiency+") OR (MH "Protocols+") OR (MH "Quality of Health Care+") OR (MH "Health Services Research+") OR (MH "Interinstitutional Relations") OR (MH "Interdepartmental Relations") OR TI Intervention* OR AB Intervention* OR TI Strateg* OR AB Strateg* OR TI Behavior OR AB Behavior OR TI Behaviour OR AB Behaviour OR TI Practice* OR AB Practice* OR TI Protocol* OR AB Protocol* OR TI Procedure* OR AB Procedure* OR TI Structure* OR AB Structure* OR TI Attitud* OR AB Attitud* OR TI Evaluat* OR AB Evaluat* OR TI Training OR AB Training OR TI Education OR AB Education OR TI Communication OR AB Communication OR TI Request* OR AB Request* OR TI Program OR AB Program OR TI Programme OR AB Programme OR TI Planning OR AB Planning OR TI Management* OR AB Management* OR TI Identif* OR AB Identif* OR TI Referral* OR AB Referral* OR TI authorization OR AB authorization OR TI authorisation OR AB authorization OR TI Consent* OR AB Consent* OR TI Decision* OR AB Decision* OR TI Efficacy OR AB Efficacy OR TI Efficiency OR AB Efficiency OR TI Satisfaction OR AB Satisfaction OR TI Knowledge OR AB Knowledge OR TI Skill* OR AB Skill* OR TI Quality OR AB Quality)

Hits: 1225.

**PsycINFO until April 24, 2019**

(Tissue donation/ OR brain damage/ OR traumatic brain injury/ Organ donor*.ti,ab. OR Potential donor*.ti,ab. OR Organ donat*.ti,ab. OR Organ procurement*.ti,ab. OR Brain death.ti,ab. OR Brain dead.ti,ab.) AND (medical personnel/ OR nurses/ OR physicians/ OR neurologists/ OR interpersonal relationships/ OR health personnel attitudes/ OR intensive care/ OR palliative care/ OR Patient care.ti,ab. OR Medical staff.ti,ab. OR Physician*.ti,ab. OR Health personnel.ti,ab. OR Nurse*.ti,ab. OR Critical Care.ti,ab. OR Intensive care.ti,ab. OR Terminal care.ti,ab. OR End-of-life care.ti,ab. OR Hospital*.ti,ab. OR Requestor*.ti,ab. OR Requester*.ti,ab. OR Organization.ti,ab. OR Organisation.ti,ab. OR Coordinator.ti,ab.) AND (interpersonal communication/ OR cooperation/ OR communication skills training/ OR personnel training/ OR medical education/ OR program development/ OR program evaluation/ OR organizational effectiveness/ OR informed consent/ OR quality of care/ OR health care services/ OR emergency services/ OR public relations/ OR Intervention*.ti,ab. OR Strateg*.ti,ab. OR Behavior.ti,ab. OR Behaviour.ti,ab. OR Practice*.ti,ab. OR Protocol*.ti,ab. OR Procedure*.ti,ab. OR Structure*.ti,ab. OR Attitud*.ti,ab. OR Evaluat*.ti,ab. OR Training.ti,ab. OR Education.ti,ab. OR Communication.ti,ab. OR Request*.ti,ab. OR Program.ti,ab. OR

Programme.ti,ab. OR Planning.ti,ab. OR Management*.ti,ab. OR Identif*.ti,ab. OR Referral*.ti,ab. OR authorization.ti,ab. OR authorisation.ti,ab. OR Consent*.ti,ab. OR Decision*.ti,ab. OR Efficacy.ti,ab. OR Efficiency.ti,ab. OR Satisfaction.ti,ab. OR Knowledge.ti,ab. OR Skill*.ti,ab. OR Quality.ti,ab.)

Hits: 707.

**Cochrane Library until April 24, 2019**

([mh "tissue and organ procurement"] OR [mh "brain death"] OR Organ donor*:ti,ab,kw OR Potential donor*:ti,ab,kw OR Organ donat*:ti,ab,kw OR Organ procurement*:ti,ab,kw OR "Brain death":ti,ab,kw OR "Brain dead":ti,ab,kw) AND ([mh "Patient Care Team"] OR [mh "Critical Care Nursing "] OR [mh "Medical staff"] OR [mh Nurses] OR [mh "Nursing staff"] OR [mh Physicians] OR [mh "Professional-family relations"] OR [mh "Attitude of Health Personnel"] OR [mh "Critical care"] OR [mh "Intensive care units"] OR [mh ^"Terminal Care"] OR “Patient care”:ti,ab,kw OR “Medical staff”:ti,ab,kw OR Physician*:ti,ab,kw OR "Health personnel":ti,ab,kw OR Nurse*:ti,ab,kw OR "Critical Care":ti,ab,kw OR "Intensive care":ti,ab,kw OR "Terminal care":ti,ab,kw OR "End-of-life care":ti,ab,kw OR Hospital*:ti,ab,kw OR Requestor*:ti,ab,kw OR Requester*:ti,ab,kw OR "Organization":ti,ab,kw OR "Organisation":ti,ab,kw OR "Coordinator":ti,ab,kw) AND ([mh Communication] OR [mh "Cooperative Behavior"] OR [mh "Hospital planning"/OA] OR [mh "Education, Professional"] OR [mh "Program development"] OR [mh "Referral and consultation"] OR [mh "Efficiency, organizational"] OR [mh "Critical Pathways"] OR [mh "Third-party consent"] OR [mh "Quality of Health Care"] OR [mh "Health Services Research"] OR [mh "Interinstitutional Relations"] OR Intervention*:ti,ab,kw OR Strateg*:ti,ab,kw OR "Behavior":ti,ab,kw OR "Behaviour":ti,ab,kw OR Practice*:ti,ab,kw OR Protocol*:ti,ab,kw OR Procedure*:ti,ab,kw OR Structure*:ti,ab,kw OR Attitud*:ti,ab,kw OR Evaluat*:ti,ab,kw OR "Training":ti,ab,kw OR "Education":ti,ab,kw OR "Communication":ti,ab,kw OR Request*:ti,ab,kw OR "Program":ti,ab,kw OR "Programme":ti,ab,kw OR "Planning":ti,ab,kw OR Management*:ti,ab,kw OR Identif*:ti,ab,kw OR Referral*:ti,ab,kw OR "Authorization":ti,ab,kw OR "authorisation":ti,ab,kw OR Consent*:ti,ab,kw OR Decision*:ti,ab,kw OR "Efficacy":ti,ab,kw OR "Efficiency":ti,ab,kw OR "Satisfaction":ti,ab,kw OR Knowledge:ti,ab,kw OR Skill*:ti,ab,kw OR "Quality":ti,ab,kw)

Hits: 638.
