# Supplementary material for: Interventions aimed at healthcare professionals to increase the number of organ donors: a systematic review
Source: Crit Care. 2019 Jun 20;23:227. doi: 10.1186/s13054-019-2509-3 (PMC6587298; doi:10.1186/s13054-019-2509-3)
Supplement: Supplementary file 2 — Data extraction form. This extraction form was used to extract the data from the included articles. (DOC 246 kb) [file 13054_2019_2509_MOESM2_ESM.doc]

# Additional file 2. Data extraction form

| 1. **Reviewers** | |
| --- | --- |
| 1. Name reviewer |  |
| 1. Date |  |
| 1. Cross-checked |  |

| 1. **Study** | |
| --- | --- |
| 1. Title |  |
| 1. Authors (>2 et al.,) |  |
| 1. Year |  |
| 1. Source |  |

| 1. **Objective and methods** | |
| --- | --- |
| 1. Objective or aim |  |
| 1. Study design |  |
| 1. Retrospective or prospective study |  |
| 1. Pilot/ feasibility study |  |
| 1. Study duration (inclusion/ recruitment) |  |

| 1. **Setting, population and sample size** | |
| --- | --- |
| 1. Country |  |
| 1. Hospital type | Beds: n= |
| 1. Number of hospitals |  |
| 1. Department |  |
| 1. Type of ICU (if applicable and known) |  |
| 1. Inclusion criteria for the study population |  |
| 1. Exclusion criteria |  |
| 1. Target population | Population Group 1:  n=  Population Group 2:  n=  Population Group 3:  n=  Population Group 4:  n= |
| 1. Drop-out rate (%) (+reasons) |  |
| 1. Tot number of patients at final analysis (Intention-To-Treat and Per-Protocol) |  |

| 1. **Intervention** | |
| --- | --- |
| 1. Description of intervention |  |
| 1. Total number of intervention groups |  |
| 1. Description of control group / usual care |  |
| 1. Information on implementation |  |
| 1. Duration intervention |  |

| 1. **Outcomes and results** | | | | |
| --- | --- | --- | --- | --- |
| 1. Quantitative outcomes   *Outcome measure; effect (mean and SD, %, rate and confidence interval, other); follow-up period (h,d, mo,yr); p-value* | Outcome measure 1:  Effect:  Follow-up:  P-value:  Outcome measure 2:  Effect:  Follow-up:  P-value:  Outcome measure 3:  Effect:  Follow-up:  P-value:  Outcome measure 4:  Effect:  Follow-up:  P-value: | | | |
|  | *Outcome measure; follow-up period* | *Effect control group* | *Effect intervention group* | *p-value* |
|  |  |  |  |  |
|  |  |  |  |  |
|  |  |  |  |  |
|  |  |  |  |  |

| 1. **Miscellaneous** | |
| --- | --- |
| 1. Key conclusions of study authors |  |
| 1. Miscellaneous comments from the reviewers |  |
