# Supplementary material for: Interventions aimed at healthcare professionals to increase the number of organ donors: a systematic review
Source: Crit Care. 2019 Jun 20;23:227. doi: 10.1186/s13054-019-2509-3 (PMC6587298; doi:10.1186/s13054-019-2509-3)
Supplement: Supplementary file 3 — Quality assessment of the included studies according to suggested risk of bias criteria for Effective Practice and Organisation of Care (EPOC) reviews [11]. This additional file shows the quality assessment of the included studies. (DOCX 16 kb) [file 13054_2019_2509_MOESM3_ESM.docx]

**Additional file 3.** Quality assessment of the included studies according to suggested risk of bias criteria for Effective Practice and Organisation of Care (EPOC) reviews [11]

| **Study, year [reference]** | **Intervention independent of other changes** | **Shape of intervention effect pre-specified** | **Intervention unlikely to affect data collection** | **Knowledge of the allocated interventions adequately prevented during the study** | **Incomplete outcome data** | **Selective outcome reporting** | **Other risks of bias** | **Random sequence generation** | **Allocation concealment** | **Baseline outcome measurements similar** | **Baseline characteristics similar** | **Protection against contamination** |
| --- | --- | --- | --- | --- | --- | --- | --- | --- | --- | --- | --- | --- |
| Adanir, et al., 2014 [13] |  |  |  | Unclear | Low | Low | High | High | Low | Unclear | Low | High |
| Beasley, et al., 1997 [17] | High | Low | High | High | Low | Low | High |  |  |  |  |  |
| Beigee, et al., 2017 [16] | High | Low | Unclear | High | Low | Low | High |  |  |  |  |  |
| Bires, 1999 [26] | High | Low | Low | Low | Low | Low | High |  |  |  |  |  |
| Bleakley, 2010 [18] | High | Low | Low | Low | High | Low | Low |  |  |  |  |  |
| Feest, et al., 1990 [19] | High | High | High | Low | High | High | High |  |  |  |  |  |
| Garside, et al., 2012 [20] | High | Low | Low | Low | High | High | High |  |  |  |  |  |
| Henderson, et al., 1998 [21] | High | Low | Low | Low | Low | Low | High |  |  |  |  |  |
| Ismail, et al., 2018 [32] | High | Low | Low | Low | Low | Low | High |  |  |  |  |  |
| Jansen, et al., 2011 [33] |  |  |  | Low | Low | Low | Low | High | Low | Low | Unclear | Low |
| Krekula, et al., 2014 [34] |  |  |  | Low | Low | Low | High | High | High | Low | High | Low |
| Lenzi, et al., 2014 [27] | High | Low | Low | Low | Low | Low | High |  |  |  |  |  |
| Linyear, et al., 1999 [22] | High | Low | Low | Low | Low | High | Low |  |  |  |  |  |
| Manyalich, et al., 2012 [28] | High | Low | Low | Low | Low | Low | Low |  |  |  |  |  |
| Mulvania, et al., 2014 [29] | High | High | Unclear | Low | Low | Low | High |  |  |  |  |  |
| Sandiumenge, et al., 2018 [25] | High | Low | Low | Low | Low | Low | High |  |  |  |  |  |
| Siminoff, et al., 2009 [30] | High | Low | Unclear | Low | Low | Low | Low |  |  |  |  |  |
| Siminoff, et al., 2015 [31] |  |  |  | Low | High | High | High | High | High | Unclear | Unclear | High |
| Stark, et al., 1994 [23] | High | Low | Unclear | Low | Low | Low | High |  |  |  |  |  |
| Von Pohle, 1996 [35] | High | Low | Low | Low | Low | Low | Low |  |  |  |  |  |
| Young, et al., 2009 [12] |  |  |  | Low | Low | Low | High | Low | Low | Unclear | High | High |
| Zier, et al., 2017 [24] | High | Low | Low | Low | Low | Low | High |  |  |  |  |  |

Two checklists were used: the risk of bias criteria for studies with a separate control group and the risk of bias criteria for interrupted times series studies (also used for studies without control group).
An empty cell means that the specific criteria is not applicable for the study.
